# The Application of Recovery Strategies in Basketball: A Worldwide Survey

**DOI:** 10.3389/fphys.2022.887507

**Published:** 2022-06-16

**Authors:** Marco Pernigoni, Daniele Conte, Julio Calleja-González, Gennaro Boccia, Marco Romagnoli, Davide Ferioli

**Affiliations:** ^1^ Department of Coaching Science, Lithuanian Sports University, Kaunas, Lithuania; ^2^ Institute of Sport Science and Innovations, Lithuanian Sports University, Kaunas, Lithuania; ^3^ Department of Physical Education and Sport, Faculty of Education and Sport, University of the Basque Country (UPV/EHU), Vitoria, Spain; ^4^ Department of Clinical and Biological Sciences, University of Turin, Turin, Italy; ^5^ Faculty of Science of Physical Education and Sport, University of Valencia, Valencia, Spain; ^6^ UCAM Research Center for High Performance Sport, Catholic University of Murcia, Murcia, Spain

**Keywords:** evidence-based practice, descriptive research, questionnaire, practitioners, team sports

## Abstract

The purpose of this study was to assess the perceived usefulness, actual use and barriers to the implementation of recovery strategies among basketball practitioners. 107 participants (strength and conditioning coaches, sport scientists, performance specialists) from different countries and competitive levels completed an online survey. Most participants rated recovery strategies as either extremely (46%) or very important (49%). Active recovery, massage, foam rolling, and stretching were perceived as most useful (80, 73, 72 and 59% of participants, respectively) and were most frequently adopted (68, 61, 72 and 67%, respectively). Participants mentioned lack of devices and facilities (51%), excessive cost (51%), lack of time (27%), players’ negative perception (25%) and lack of sufficient evidence (16%) as barriers to the implementation of recovery strategies. The present findings reveal that some dissociation between scientific evidence and perceived effectiveness was present among the study participants. A possible solution would be to ensure that scientific evidence-based guidelines are followed when considering the application of recovery strategies. Regarding actual use, participants favored easily implementable strategies (e.g. active recovery, stretching), rather than evidence-supported, but expensive and/or impractical strategies (e.g. whole-body cryotherapy). Possible solutions may include the use of practical tools that don’t need specific facilities, the development and validation of new low-cost recovery devices, the promotion of players education regarding recovery strategies, and conducting further research to increase the scientific knowledge in the area.

## Introduction

Basketball is a popular intermittent court-based team sport, which requires players to perform repeated high-intensity activities, including sprinting, shuffling, jumping, accelerations, decelerations and changes of direction (Stojanović et al., 2018; Pernigoni et al., 2021). The nature of basketball activity places the athlete’s body under stress and can cause a disruption of homeostasis, resulting in exercise-induced muscle damage and delayed onset of muscle soreness, reduced range of motion, impaired kinesthetic awareness, inflammatory and immunological responses (Chatzinikolaou et al., 2014; Doma et al., 2018; Moreira et al., 2018). The short-term effects of a single session, although necessary for long-term adaptation, may have a negative influence on performance lasting up to 48 h (Chatzinikolaou et al., 2014; Pliauga et al., 2015). This transitory situation is especially relevant when considering that professional teams often face congested schedules with multiple games played in close succession, such as during the playoff period (Ferioli et al., 2021). Thus, the implementation of strategies limiting the negative consequences of basketball activity and improving the time course of recovery is crucial to allow players to train and perform effectively when the time between sessions is short (Dupuy et al., 2018; Huyghe et al., 2020).

The use of post-exercise recovery strategies such as massage, compression garments, hydrotherapy, cryotherapy, stretching, foam rolling, sleep and nutritional approaches has been extensively investigated in various sports ( Herbert et al., 2011; Poppendieck et al., 2016; Rose et al., 2017 ; Dupuy et al., 2018; Wiewelhove et al., 2019; Ortiz et al., 2019 ; Bongiovanni et al., 2020; Davis et al., 2020; Skinner et al., 2020; Afonso et al., 2021; Kwiecien and McHugh, 2021). However, only a relatively limited number of studies has specifically analyzed the efficacy of such interventions in basketball (Calleja-González et al., 2016; Huyghe et al., 2020; Davis et al., 2021). Therefore, basketball practitioners are often not provided with specific evidence-based guidelines and it is unknown how such research is considered and implemented. To the best of the authors’ knowledge, there are no studies available to describe which recovery strategies are currently used by basketball practitioners from different countries and competitive levels. Assessing the interventions employed in a real-world context can provide useful information about how the scientific principles supporting recovery strategies are applied, thus understanding the factors that may limit the use of certain tools and methods. Such insight would provide practitioners with relevant information on recovery strategies used in basketball, assist the development of future research on the topic, and could help find solutions for a better and more efficient translation of scientific evidence to daily practice.

Therefore, the aims of the current study were to assess the perceived usefulness and actual use of recovery strategies among basketball practitioners, and to identify potential barriers which may prevent the use of recovery strategies in real-world contexts. It was hypothesized that practitioners’ perceived usefulness would be in accordance with established scientific evidence. Regarding actual use and potential barriers, practitioners were expected to favor practical and inexpensive strategies, due to greater availability and easier implementation.

## Materials and Methods

### Participants

A freely accessible online questionnaire was designed and subsequently advertised via e-mail, phone or social media to gather information about the perception and use of recovery strategies among basketball practitioners. The survey remained available online for approximately 2 months (i.e., from May 22nd until July 26th, 2020). At the end of this period, a convenient sample size of 107 practitioners completed the survey. As this is a pilot study, the a priori sample size calculation was not performed. We considered the amount of responders to be adequate in relation to the sample size of previous similar studies ( Van Wyk and Lambert, 2009; Fleming et al., 2018; Field et al., 2021 ). The study was approved by the Lithuanian Sports University Ethics Committee [2020-05-19 NR. BNL-TRS(B)-2020-304] and was designed according to the Declaration of Helsinki. Participants were informed regarding the study aims in advance and participation was voluntary.

### Design

A descriptive design was used for this study. An online survey (https://docs.google.com/forms/d/e/1FAIpQLScKLB55hk3UzjSVuPLq1jc1cT7AhSXG9hPwcE4I8zLjgdqSUQ/viewform) including a combination of multiple choice (i.e. only one answer allowed), checkboxes (i.e. multiple answers allowed), Likert scales and open-ended, free-text responses were used to identify perceived usefulness and actual use of various recovery strategies among basketball practitioners. A total of 17 questions were organized in three distinct sections: sociodemographic data (8 questions), perceived usefulness (3 questions) and actual use (6 questions) of recovery strategies. The questionnaire was designed in a user-friendly manner, with completion only requiring about 5 minutes.

### Methodology

The first section (sociodemographic data) was designed to receive information about the age, gender, experience, qualifications (i.e. academic degrees or other professional certifications), and role of the participants within their basketball team. In addition, the gender and competitive level of the team were assessed in this section. The main aim of the second section (perceived usefulness) was to investigate whether participants generally perceived recovery strategies as important, the reasoning behind this assumption, and which strategies they believed to have a beneficial effect. The third section (actual use) encompassed questions about the frequency, timing, place and type of recovery interventions implemented by the participants, as well as potential barriers which prevent them from utilizing one or more of these strategies. Where pertinent, multiple choice and checkbox questions were designed so that participants could add further answers (i.e. “other”), which were not included in the available options. One open-ended, free-text question was included at the end of the survey, allowing participants to add further comments, suggestions or feedbacks.

### Statistical Analysis

As detailed in previous research (Starling and Lambert, 2018; Altarriba-Bartes et al., 2020), absolute and relative (percentage) frequencies were used for the categorical variables, and qualitative terms were used to characterize the observed frequencies as follows: All = 100% of participants; Most = ≥75%; Majority = 55–75%; Approximately half = ∼50%; Approximately a third = ∼30%; Minority = <30%. Furthermore, 90% compatibility limits were calculated for each proportion (i.e. the percentage of participants selecting a given answer to a given question) to assess its margin of error, using the following formula: 90% compatibility limits = ± 1.65*√[x (100-x)/n], where x is the proportion and n is the total sample size. Data were extracted from the online survey (Google Forms^®^) and downloaded onto a spreadsheet (Microsoft^®^ Excel for Windows version 16.0.13929.20206) for analysis.

## Results

### Sociodemographic Data

The sociodemographic characteristics of the participants are reported in the Supplementary material S1. Most of them were males [86% (90% compatibility limits ±6%)], aged between 21 and 40 years old [74% (±7%)] and working with male teams [78% (±7%)]. Most participants [79% (±6%)] were employed as strength and conditioning coaches and held an academic degree in sport sciences, either Bachelor [38% (±8%)] or Master [30% (±7%)]. Moreover, most participants were part of high-level teams, as 38% (±8%) of them were working in first division clubs, 17% (±6%) in second division clubs, 13% (±5%) with the youth academy of a national-level club, and 7% (±4%) with national senior teams.

### Perceived Usefulness and Actual Use of Recovery Strategies

**FIGURE 1:**
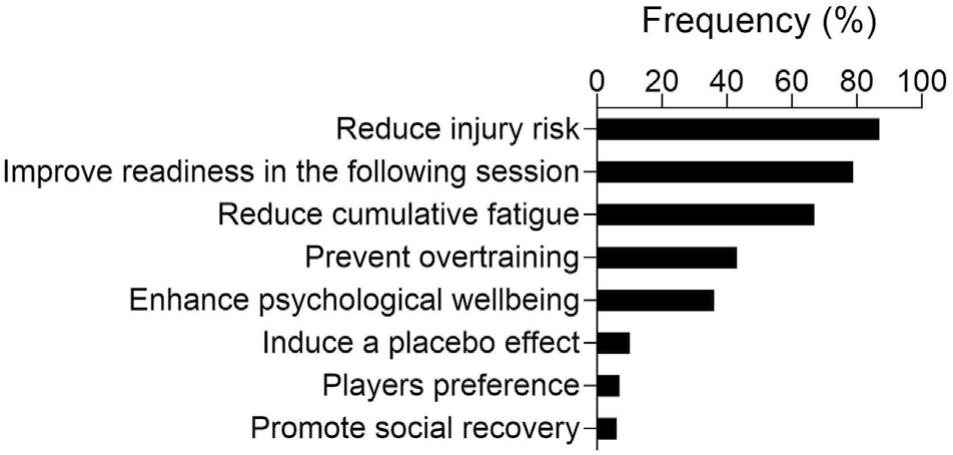
Reported relative frequencies for the rationale justifying the adoption of recovery strategies. Answers are listed in descending order, from the most to the least frequent.

Most participants rated recovery strategies as either extremely important [46% (±8%)] or very important [49% (±8%)]. As displayed in Figure 1, the most frequent reasons for adopting recovery strategies were to reduce injury risk [87% (±5%)], increase readiness for the following practice session or game [79% (±6%)], and reduce cumulative fatigue [67% (±7%)].

**FIGURE 2:**
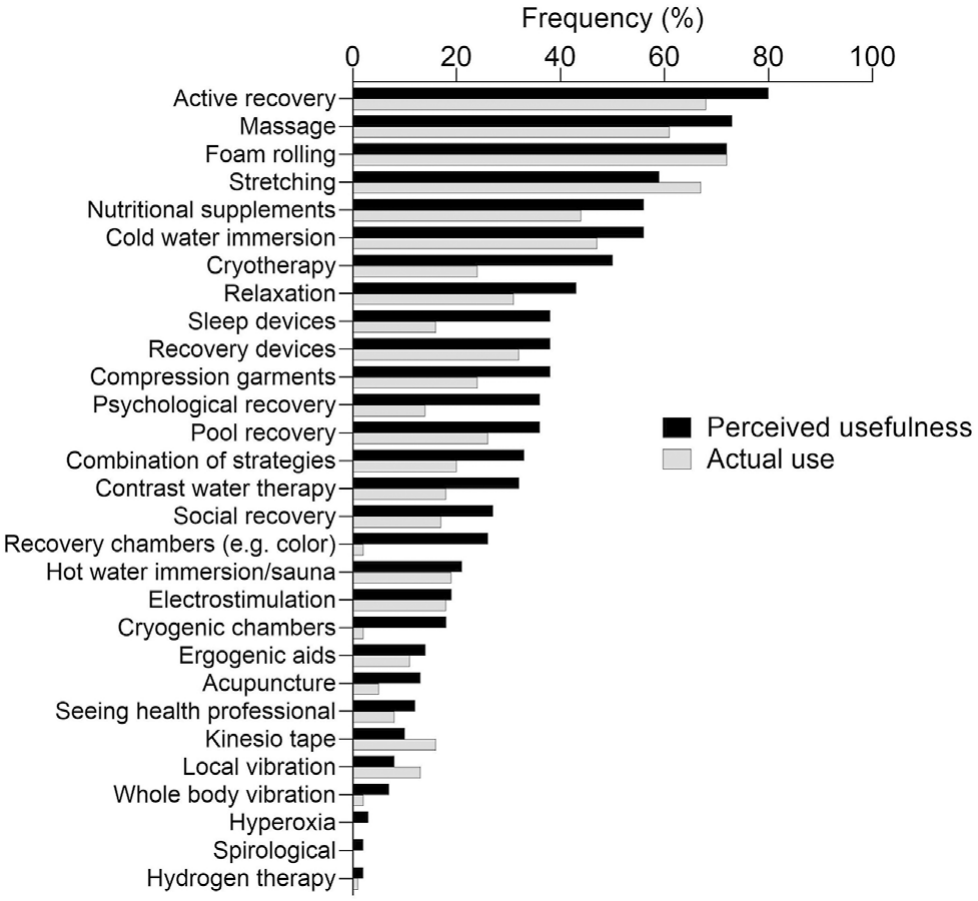
Reported relative frequencies for perceived usefulness and actual use of recovery strategies. Strategies are listed in descending order, from the most to the least perceived as useful.

The frequencies for perceived usefulness and actual use are reported in Figure 2. Briefly, active recovery, massage, foam rolling and stretching were the strategies perceived as most useful and most frequently adopted. Cryotherapy, recovery chambers, sleep devices, and psychological interventions showed the greatest discrepancy between perceived usefulness and actual use. When asked about the frequency of use, 15% (±6%) of the participants declared that they always made use of recovery strategies, 19% (±6%) usually, 29% (±7%) frequently, 23% (±7%) sometimes, 10% (±5%) occasionally, 3% (±3%) rarely, while one participant [1% (±2%)] never used recovery strategies. Most of the participants made use of recovery strategies immediately after a training session [81% (±6%)] or competitive game [79% (±6%)], approximately a third of them when travelling [37% (±8%)] or in separate sessions [30% (±7%)], while a minority used them pre-training [25% (±7%)] or pre-game [21% (±7%)]. Most of the participants [89% (±5%)] reported using recovery strategies at their own sports hall (i.e., where the basketball court is located), approximately half of them at home [48% (±8%)] or at hotels [46% (±8%)], while a minority used them at other (i.e., guest) arenas/gyms [27% (±7%)], mobility units [20% (±6%)] and while travelling (e.g., bus, plane) [20% (±6%)].

**FIGURE 3:**
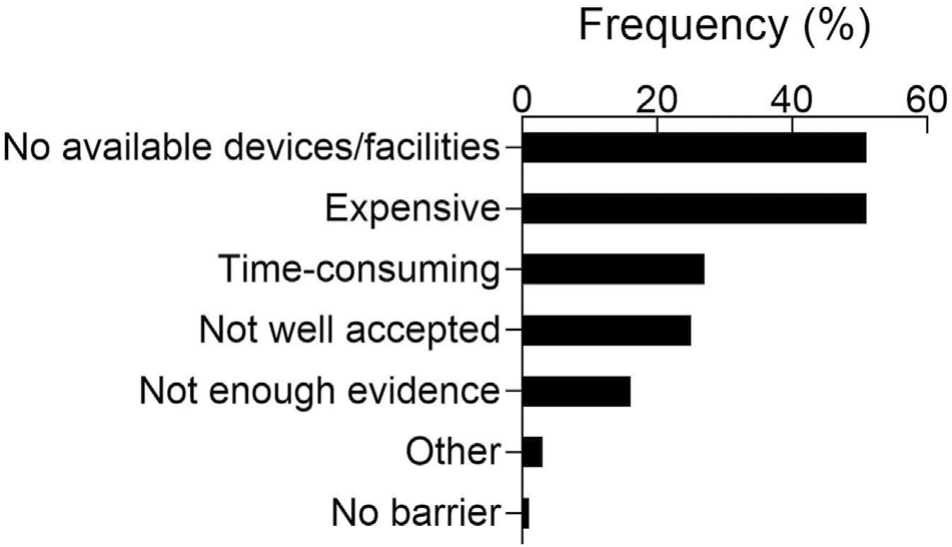
Reported relative frequencies for perceived barriers to the implementation of recovery strategies. Answers are listed in descending order, from the most to the least frequent.

Finally, barriers to the implementation of recovery strategies are reported in Figure 3. Lack of devices/facilities and excessive cost were the most frequently reported barriers, with each answer being mentioned by 51% (±8%) of the participants.

## Discussion

The present study provides novel insights into the perceived usefulness and actual use of recovery strategies among basketball practitioners, while identifying the potential barriers which may prevent the use of recovery strategies in real-world contexts. Overall, nearly all participants acknowledged the importance of recovery strategies in basketball, with active recovery, massage, foam rolling and stretching being the most positively perceived and most applied strategies among basketball practitioners. Reducing injury risk, increasing the readiness for the following practice session or game, and reducing cumulative fatigue were the most popular reasons for adopting recovery strategies. With regards to barriers preventing the use of recovery strategies, the lack of specific devices/facilities and the excessive cost for their implementation were the most frequently reported.

The results of the present survey suggest that active recovery, massage, foam rolling, stretching, cold water immersion (CWI) and nutritional supplements were positively perceived by the majority of participants. While some of these strategies have shown potential for enhancing the recovery process, others are not well supported in literature or have produced mixed results. CWI has been reported to positively affect the recovery status of basketball players in terms of physical performance, muscle soreness, exercise-induced muscle damage and inflammatory markers (Montgomery et al., 2008; Delextrat et al., 2013; Sánchez–Ureña et al., 2017; Seco-Calvo et al., 2020), thus supporting its application in a real-world context. Similarly, a recent review suggested that athletes may benefit from the consumption of nutritional supplements such as vitamin D, Omega-3 fatty acids, tart cherries, beetroot and pomegranate juice to enhance the recovery process (Bongiovanni et al., 2020). In line with this, foam rolling has also shown potential benefits on sprint and strength performance, and muscle soreness in athletes, although its underlying mechanisms are largely unknown (Wiewelhove et al., 2019; Skinner et al., 2020). On the other hand, research in athletic populations has shown that active recovery might have a positive effect on performance and muscle soreness during a short period after exercise (Dupuy et al., 2018; Ortiz et al., 2019; Huyghe et al., 2020). However, the missing guidelines in terms of optimal outcome variables, intensity, duration and individualization of active recovery interventions have made it challenging to draw conclusions about the actual efficacy of this strategy (Ortiz et al., 2019). Massage has also been suggested as a beneficial method for perceptual measures of recovery (Delextrat et al., 2013; Delextrat et al., 2014; Dupuy et al., 2018; Davis et al., 2020), performance (Delextrat et al., 2014; Poppendieck et al., 2016), heart rate variability (Kaesaman, 2019), sympathetic-parasympathetic balance (Kaesaman, 2019) and blood markers (Dupuy et al., 2018). However, its effects on performance have been reported to be small or inconsistent (Poppendieck et al., 2016), whereas its impact on soreness and fatigue may be susceptible to placebo effects or biased subjective assessment (Davis et al., 2020). Finally, no beneficial effects were generally reported following stretching, with respect to post-exercise recovery (Herbert et al., 2011; Dupuy et al., 2018). As such, considering the present findings, a dissociation between established evidence and perceived effectiveness was present for the participants in the present study. Accordingly, strategies which have shown potential beneficial effects, such as whole-body cryotherapy (Rose et al., 2017; Dupuy et al., 2018; Kwiecien and McHugh, 2021), have been perceived as less effective by the participants. As previously suggested (Fullagar et al., 2019), sport practitioners may favor one-on-one or group conversations over journal articles as a source of knowledge, which could partly explain the current findings. Furthermore, it has been reported that practitioners’ perception of recovery strategies can be influenced by past experiences, according to their own feelings or players’ perception of their efficacy (Simjanovic et al., 2009). This is a potential issue for the optimization of players’ recovery, as belief-based practice—which is not always supported by scientific evidence—may result in the use of inadequate interventions for recovery purposes.

In the present study, foam rolling, active recovery, stretching and massage were the most utilized recovery strategies. These findings are not surprising, as massage is extremely popular (Davis et al., 2020), while the other mentioned strategies are practical, easy to use and do not require expensive equipment. Indeed, it has been suggested that the choice of recovery strategies could be affected by practicality and accessibility, as teams may use recovery strategies which lack robust scientific evidence, but are easily implementable (Crowther et al., 2017; Altarriba-Bartes et al., 2020). Accordingly, participants reported that the excessive cost and lack of devices/facilities were the main barriers to the use of recovery strategies. These findings are in agreement with previous research (Simjanovic et al., 2009; Rey et al., 2018; Field et al., 2021), as accessibility has been frequently reported as a barrier to the implementation of recovery strategies. Therefore, it was expected that recovery strategies which are expensive and/or require specific facilities (e.g., cryotherapy, pool recovery, contrast water therapy and recovery/sleep devices) would be used relatively rarely, despite being often perceived as useful. Specifically, accessibility could be a major obstacle when playing away from home, as dedicated spaces and materials are often not available to teams (Rey et al., 2018; Altarriba-Bartes et al., 2020). Somewhat surprisingly, only a minority (27%) of the participants indicated lack of time as a barrier, while previous studies in other sports identified it as a major obstacle (Simjanovic et al., 2009; Rey et al., 2018; Fullagar et al., 2019). Players’ negative perception of recovery strategies was mentioned as a possible barrier by a similar proportion of the participants (25%), supporting the notion that players preferences may not match scientific recommendations (Crowther et al., 2017; Field et al., 2021). Efficient communication and education of players about the methods, tools and rationale supporting evidence-based interventions is crucial in order to effectively implement sound, effective practice (Le Meur and Torres-Ronda, 2019; Field et al., 2021). Moreover, practitioners should consider minimizing the stress induced by the use of recovery strategies. This is crucial, as players are already exposed to potential stressors such as a congested competition calendar, travelling, public pressure and injuries, which may all have detrimental effects on their well-being (Jukić et al., 2018). Finally, some participants (16%) felt like scientific evidence is not solid enough to justify the use of recovery strategies. While it is true that some methods (e.g. CWI, whole-body cryotherapy, nutritional supplements) have been studied and appear promising (Rose et al., 2017; Dupuy et al., 2018; Bongiovanni et al., 2020; Kwiecien and McHugh, 2021), there is still a great number of tools which have not been sufficiently investigated or whose evidence is conflicting. Therefore, it is understandable that some practitioners would rather exploit the available time prioritizing other aspects of the training process.

Some limitations of the present study should be considered. The results of the current survey should be interpreted with caution, as sample size is limited and uncertainties for comparisons of proportions are in some cases too large to draw confident conclusions. Indeed, the minimum number of participants to be recruited in the survey was set in accordance with previous research on the topic ( Van Wyk and Lambert, 2009; Fleming et al., 2018; Field et al., 2021 ). However, the compatibility limits of proportions seem in some cases not adequate for comparison of proportions, thus suggesting a bigger sample size would be required. Furthermore, the survey was designed to be openly accessible to basketball practitioners, and was advertised through different platforms (email, phone, social media) with any interested basketball practitioner free to complete it. This recruiting strategy limited the authors in retrieving relevant and detailed information about the practitioners who took part (and those who did not) to the survey (e.g., rate of participation or refusal according to each category). In addition, the survey was only designed using the English language, potentially excluding practitioners from areas where basketball is quickly developing (e.g., some Asian countries). As such, our study was likely affected by a sample bias towards English-speaking practitioners willing to participate in the online survey and/or interested in recovery strategies. In line with these limitations, the results of present investigation do not necessarily apply to the average potential user of recovery strategies in basketball. Future studies recruiting a greater number of practitioners to overcome sample size issue and defining a better recruitment strategy to overcome possible sampling bias are warranted to draw more pertinent and reliable conclusions on this topic. For a better interpretation of the study results, it should be also considered that female teams were underrepresented (only 22% of the participants) compared to male teams (78%) and financial resources, available facilities or season schedule may vary considerably depending on country and competitive level, thus affecting the application of recovery strategies. Finally, no practitioners from the National Basketball Association participated in this study, meaning that no data was available regarding the perceived usefulness, actual use and barriers to the implementation of recovery strategies at the highest competition level in the world.

## Practical Applications

The present findings suggest that a certain degree of dissociation existed between scientific evidence and perceived effectiveness of recovery strategies among the study participants. Whenever possible, scientific evidence-based guidelines should be followed to make sure that appropriate and effective recovery interventions are considered for implementation. As for actual use, multiple barriers were identified by the participants, indicating that the selection of recovery strategies is no easy feat. Possible solutions may include choosing practical tools which could also be used while on the road and/or travelling (e.g. foam rolling), developing low-cost valid instruments to increase accessibility, promoting players education and improving communication regarding the effectiveness of recovery strategies, conducting further high-quality research to increase scientific knowledge and validate emerging tools and methods.

## Conclusion

To the best of the authors’ knowledge, this is the first study to investigate the perceived effectiveness, actual use and barriers to the implementation of recovery strategies among basketball practitioners from different countries and competitive levels. The majority of participants positively perceived active recovery, massage, foam rolling, stretching, CWI and nutritional supplements, even though some of these strategies are not conclusively supported by scientific evidence. Therefore, it appears as if a certain degree of dissociation between established evidence and perceived effectiveness was present, which may be a potential issue for the optimization of players’ recovery. As for actual use, only foam rolling, active recovery, stretching and massage were utilized by a majority of the participants in the present study. This may be a result of a lack of practicality and accessibility of other methods, as teams typically favor strategies which are easily implementable (e.g., active recovery, stretching), rather than evidence-based, but expensive and/or impractical, ones (e.g., whole-body cryotherapy). Other identified barriers included players’ negative perception of recovery strategies and lack of robust evidence in favor of certain recovery strategies, which highlights the need for better communication, players education and further research on the topic of recovery strategies.

## Data Availability

The original contributions presented in the study are included in the article/Supplementary Materials. Further inquiries can be directed to the corresponding author.
