# Supplementary material for: The Application of Recovery Strategies in Basketball: A Worldwide Survey
Source: Front Physiol. 2022 Jun 16;13:887507. doi: 10.3389/fphys.2022.887507 (PMC9243427; doi:10.3389/fphys.2022.887507)
Supplement: Supplementary file 1 [file Table1.DOCX]

Supplementary Material

Tables reporting the participants sociodemographic characteristics and the relative frequency of response (out of 107 participants).

| **Age** | **%** |
| --- | --- |
| <21 | 2 |
| 21-30 | 42 |
| 31-40 | 32 |
| 41-50 | 20 |
| 51-60 | 4 |
| >60 | 1 |

| **Gender** | **%** |
| --- | --- |
| Male | 86 |
| Female | 13 |
| Not specified | 1 |

| **Team gender** | **%** |
| --- | --- |
| Male | 78 |
| Female | 22 |

| **Role** | **%** |
| --- | --- |
| Strength and conditioning coach | 79 |
| Head of performance | 15 |
| Sport Scientist | 8 |
| Coach | 5 |
| Physiotherapist | 4 |
| Data Scientist | 1 |
| Other | 1 |

| **Experience** | **%** |
| --- | --- |
| <5 | 38 |
| 5 to 10 | 27 |
| 11 to 15 | 16 |
| 16 to 20 | 9 |
| >20 | 9 |

| **Degree** | **%** |
| --- | --- |
| Bachelor | 38 |
| NSCA/other | 32 |
| Master | 30 |
| PhD | 16 |
| Degree in other discipline | 9 |
| No degree | 4 |
| Other | 3 |

| **Country** | **%** | |  |
| --- | --- | --- | --- |
| Argentina | 1 | |  |
| Australia | 1 | |  |
| Brazil | 8 | |  |
| Canada | 1 | |  |
| China | 1 | |  |
| France | 3 | |  |
| Germany | 3 | |  |
| Israel | 1 | |  |
| Italy | 36 | |  |
| Kosovo | 1 | |  |
| Lithuania | 15 | |  |
| Macedonia | 1 | |  |
| Netherlands | 1 | |  |
| Philippines | 2 | |  |
| Serbia | 4 | |  |
| Slovakia | 1 | |  |
| Spain | 8 | |  |
| Tunisia | 1 | |  |
| Turkey | 3 | |  |
| UK | 2 | |  |
| USA | 4 | |  |
| Not specified | 5 | |  |
| **Competitive level** | | **%** | |
| 1st division club | | 38 | |
| 2nd division club | | 17 | |
| Youth academy of national-level club | | 13 | |
| 3rd division club | | 11 | |
| National senior team | | 7 | |
| Youth academy of regional-level club | | 5 | |
| Other | | 5 | |
| National youth team | | 2 | |
| College/University | | 2 | |
